# Botulinum Toxin for Drooling in Adults with Diseases of the Central Nervous System: A Meta-Analysis

**DOI:** 10.3390/healthcare11131956

**Published:** 2023-07-06

**Authors:** Chih-Rung Chen, Yu-Chi Su, Hui-Chuan Chen, Yu-Ching Lin

**Affiliations:** 1 School of Medicine, College of Medicine, National Cheng Kung University, Tainan 701401, Taiwan; i54085318@gs.ncku.edu.tw; 2 Department of Physical Medicine and Rehabilitation, National Cheng Kung University Hospital, College of Medicine, National Cheng Kung University, Tainan 704302, Taiwan; n106625@mail.hosp.ncku.edu.tw; 3 Department of Physical Medicine and Rehabilitation, College of Medicine, National Cheng Kung University, Tainan 701401, Taiwan

**Keywords:** botulinum toxins, drooling, central nervous system diseases, meta-analysis

## Abstract

(1) Background: The purpose of this study was to determine whether the drooling of adult patients with diverse central nervous system diseases can be treated with botulinum toxin type A. (2) Methods: The Cochrane Library, MEDLINE, and Embase were all searched for studies that fit the inclusion criteria. The patients in the studies had to be adults (>18 years old), and the studies had to be randomized placebo-controlled trials, controlled trials, or prospective studies. Each study had to have enough quantifiable data available for meta-analysis. The primary outcome measure was the Drooling Severity and Frequency Scale (DSFS). (3) Results: The meta-analysis comprised three studies. A statistically significant difference in DSFS score between the treatment and control groups was observed in the meta-analysis, with an overall standardized mean difference of −0.9377 (95% CI, −1.2919 to −0.5836; *p* < 0.0001). A total of seven studies were ineligible for inclusion in the meta-analysis and were only assessed as qualitative data. All qualitative studies showed a significant reduction in DSFS score a few weeks or months after the injection of botulinum toxin. (4) Conclusions: Botulinum toxin type A is safe and effective as a treatment for drooling in adult patients with central nervous system diseases.

## 1. Introduction

Drooling has been commonly found in patients with diseases in the central nervous system such as Parkinson’s disease, amyotrophic lateral sclerosis (ALS), and cerebral palsy [1,2,3]. Numerous options have been advocated to manage drooling, but none of them are universally successful [4,5]. Anticholinergic medications such as glycopyrrolate and scopolamine have been used to control drooling, but they produce a few side effects such as dizziness, headaches, restlessness, irritability, hyperactivity, constipation, facial flushing, thick mucoid secretions, dehydration, urinary retention, dilated pupils, slight visual impairment, and seizures [5,6,7]. Surgeries that alter existing neural pathways for salivation may alleviate the severity of drooling, but these operations usually cause dry mouth and other complications [8]. Botulinum toxin (BoNT) injections into the salivary glands have been studied as an alternative for drooling in patients with central nervous system diseases. Injected BoNT will be endocytosed into the neuron and start the proteolytic cleavage of proteins necessary for synaptic transmission, thus inhibiting nerve signaling [9].

The two types of botulinum toxin commonly used in clinical settings are type A and B botulinum toxins. Botulinum toxin type A (BoNT-A) is the one most studied and the most frequently injected [10,11]. Due to the small numbers of participants in various experiments, we aimed to perform a systematic review and meta-analysis to evaluate the effectiveness and the recommended dosage of BoNT-A for the management of drooling in adult patients with central nervous system diseases.

## 2. Materials and Methods

***Literature Search:*** This systemic review and meta-analysis involved the use of the following online databases of published research: MEDLINE, Embase, and the Cochrane Library. For MEDLINE, the search syntax used was (exp * Botulinum Toxins/or exp * Botulinum Toxins, Type A/) and exp * Sialorrhea/and exp *Central Nervous System Diseases/. For Embase, the search syntax used was ‘botulinum toxin’/exp AND ‘hypersalivation’/exp AND ‘central nervous system disease’/exp. For the Cochrane Database of Systematic Reviews and the Cochrane Central Register of Controlled Trials, the search syntax used was (“botulinum toxins”[MeSH Terms]) AND (“sialorrhea”[MeSH Terms]) AND (“central nervous system diseases”[MeSH Terms]).

***Inclusion Criteria:*** The objective of this meta-analysis was to evaluate the effectiveness of BoNT-A in reducing the severity and frequency of drooling in patients with central nervous system diseases. Therefore, this meta-analysis employed the following inclusion criteria: (1) the research included had to use botulinum toxin type A as their treatment group(s); (2) the participants had to have central nervous system diseases; (3) the included studies had to be randomized placebo-controlled trials, controlled trials, or prospective studies; (4) the articles had to be published in English; (5) the participants involved in the included studies had to be older than 18 years old; and lastly, (6) there had to be sufficient quantifiable data available for meta-analysis.

Different outcome measures were used to assess the severity, frequency, and impact of drooling on patients’ daily lives. Popular subjective assessment methods included the visual analogue scale, the Teacher Drooling Scale, the Drooling Impact Scale, the Drooling Quotient, the Drooling Severity and Frequency Scale (DSFS), etc. [12,13,14]. Objective assessment methods included the number of paper handkerchiefs or towels used during the time of assessment and saliva weight measured using cups that were positioned near the oral region [4]. During the literature search, the Drooling Severity and Frequency Scale (DSFS) created by Thomas-Stonell and Greenberg was found to be one of the most common outcome measures when assessing the severity and frequency of drooling. Therefore, the DSFS was the only outcome measure used for this meta-analysis. Table 1 shows how patients are assessed and given a score according to the severity and the frequency of their drooling.

## 3. Results

### 3.1. Study Characteristics

**Figure 1:**
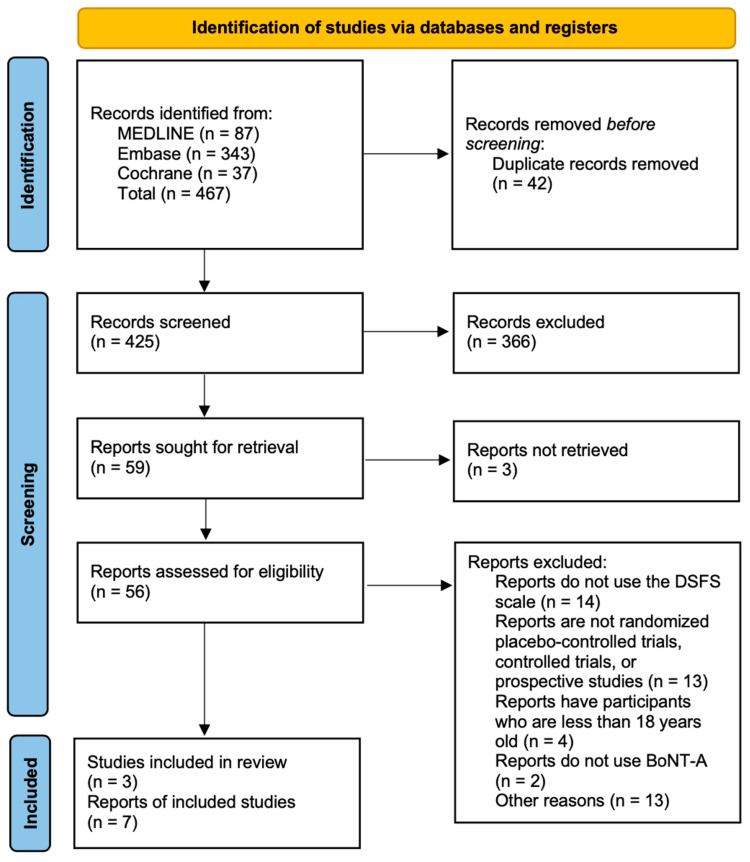
Flow diagram showing the number of studies selected for this study.

A total of three randomized controlled trials were included in the meta-analysis [15,16,17]. Seven other studies were discussed as a systematic review since they did not have any control group and only had baseline and posttreatment data for the treatment group(s). Figure 1 shows the flow diagram for the identification of eligible studies. Table 2 and Table 3 show the different characteristics of each study included for the meta-analysis and the systematic review, respectively.

### 3.2. Meta-Analysis

A total of three studies were included in the meta-analysis [15,16,17]. Narayanaswami et al. published their article on Parkinsonism and related disorders examining the efficacy of incobotulinum toxin A (Xeomin) as a treatment of sialorrhea in Parkinson’s disease through a double-blind, randomized, placebo-controlled trial [15]. One hundred units of incobotulinum toxin A was injected into both sides of the parotid and submandibular glands, whereas the control group received saline as a placebo. This study did not demonstrate efficacy of incobotulinum toxin A for drooling in Parkinson’s disease. However, the authors performed a meta-analysis on the same topic, included five studies, and discovered that their meta-analysis showed a significant reduction in treatment groups receiving botulinum toxin, but also a high degree of heterogeneity.

In a double-blind, placebo-controlled trial from Mancini et al., a total of 450 units of abobotulinum toxin A (Dysport) was injected into each of the ten patients in the treatment group, and the control group received saline as a placebo [16]. A Wilcoxon two-sample test calculating between-group comparisons showed that there was a statistically significant difference between the two groups one week after administration (*p* = 0.005). However, the DSFS score started returning to the baseline value two to three months after injection.

The double-blind, randomized, placebo-controlled trial from Jost et al. had the largest number of participants in all studies; 184 patients participated in the study, and 11 of them discontinued the study [17]. The patients were randomly assigned to either the control group or one of the two treatment groups (receiving either 75 U or 100 U of incobotulinum toxin A). There was a significant reduction in sialorrhea severity and frequency and saliva production in all outcome measures. The DSFS score was reduced in both treatment groups at four, eight, and twelve weeks after injection.

**Figure 2:**
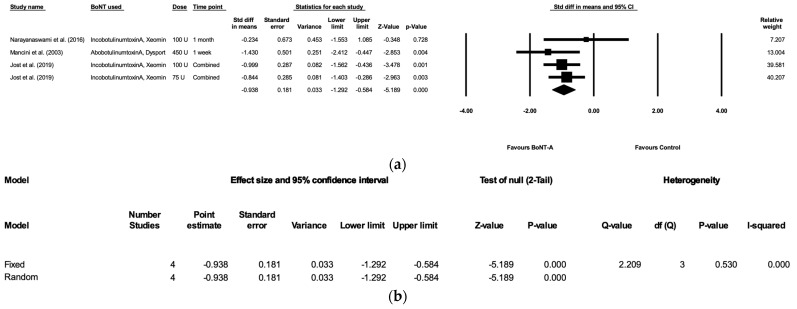
(**a**) Forest plot of the four randomized, placebo-controlled trials included for this meta-analysis [15,16,17]. The random effects model is used. Each treatment group with its respective dosage is studied separately. The time points of each study are combined as a mean and are not interpreted as independent groups. A statistically significant improvement in DSFS score was observed, with an overall standardized difference in means of −0.938 (95% CI, −1.292 to −0.584; *p* < 0.0001). (**b**) Heterogeneity is assessed with the *I*^2^ statistic (0%). Moderate or high heterogeneity in this meta-analysis is unlikely. The forest plot and the effect size and measure are created and calculated using Comprehensive Meta-Analysis software.

Figure 2 shows the forest plot of the three randomized, placebo-controlled trials included in this meta-analysis using Comprehensive Meta-Analysis software (Biostat Inc. NJ, USA). Since between-study variability may be present, this meta-analysis used a random effects model and standardized differences with 95% confidence intervals (CI). The result showed a statistically significant improvement in DSFS score with an overall standardized mean difference of −0.9377 (95% CI, −1.2919 to −0.5836; *p* < 0.0001). The *I*^2^ statistic is 0%, which suggests that moderate or high heterogeneity in this meta-analysis is unlikely.

### 3.3. Risk of Bias Assessment

**Figure 3:**
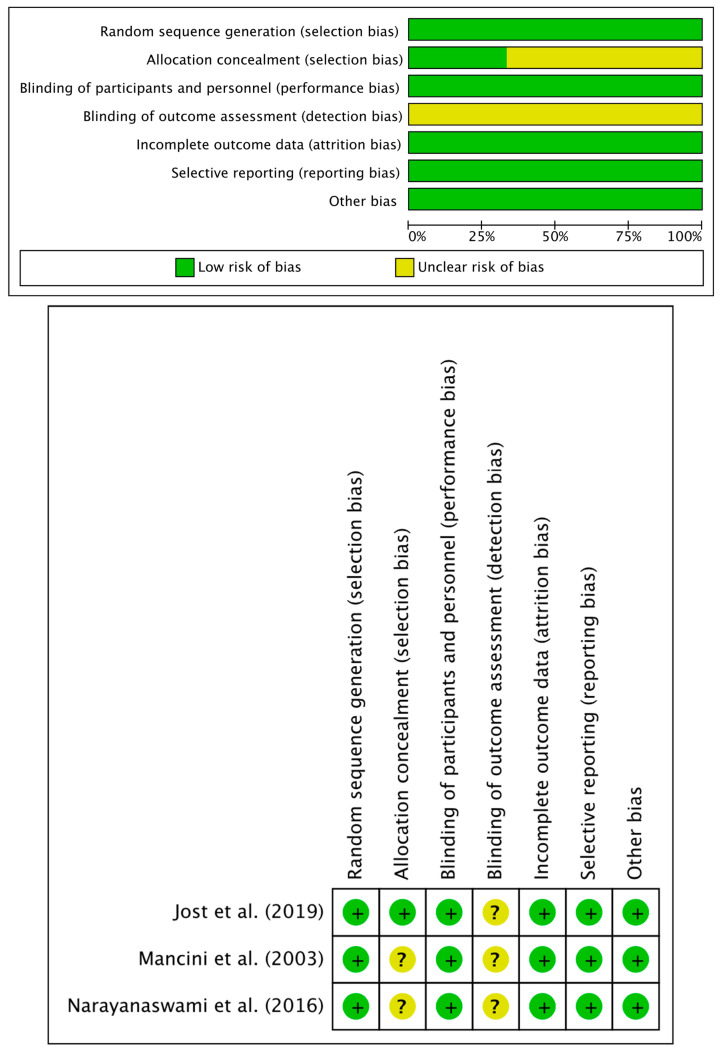
Risk of bias graph and summary [15,16,17].

Cochrane’s risk of bias graph and a summary table were created to assess whether different biases existed in each study included in the meta-analysis (Figure 3). All three studies reported a low risk of random sequence allocation bias, performance bias, attrition bias, and reporting bias [15,16,17]. Two of the studies did not report any information regarding allocation concealment [15,16]. All three studies did not mention any blinding of outcome assessment [15,16,17].

### 3.4. Systematic Review

Several other studies investigated the efficacy of BoNT-A in reducing the symptoms of drooling. However, most of the relevant studies found in online databases did not have a placebo-controlled group. Nevertheless, these studies proved that there was a significant change in drooling severity and frequency before and after injecting BoNT-A.

Three research articles with only treatment groups studied the efficacy of BoNT-A on sialorrhea in Parkinson’s disease [18,19]. Nóbrega et al. studied the efficacy of 250 U (125 U given in two locations of the parotid gland) of abobotulinum toxin A (Dysport) for drooling in patients with central nervous system diseases [18]. Out of a total of 21 patients, 18 patients experienced a decrease in drooling severity, whereas only 8 patients noticed a difference in the frequency. Adverse events were found in three patients; two of them developed dry mouth lasting for a month, and the other experienced bilateral local edema. Nonetheless, the study reported that the changes before the injection of abobotulinum toxin A and a month after the injection were statistically significant (*p* < 0.001) [18].

Pal et al. also published a study examining the efficacy of onabotulinum toxin A (Botox) as a treatment for severe drooling in patients with Parkinson’s disease [19]. Baseline DSFS score was recorded, and eight weeks after the injection of 15 units of onabotulinum toxin A at both sides of the parotid glands, one patient experienced a significant improvement in both the severity and frequency of drooling. However, one patient did not show significant improvement after the injection. Seven other patients went through the same process mentioned above, but also participated further in the experiment by being injected with 30 units of onabotulinum toxin A at both sides of the parotid glands. Two patients experienced a significant change in the DSFS score after the injection, three patients only experienced slight improvement, and two patients did not report a reduction in the severity or frequency of drooling. Additionally, the study provided data on the saliva weight of each patient as another outcome measure, and there was a statistically significant difference between baseline and post-injection values (*p* < 0.01) [19].

Santamato et al. performed research with a similar objective and showed that the injection of onabotulinum toxin A led to a significant reduction in the DSFS score from the baseline value [20]. A total of 18 patients with Parkinson’s disease were injected with approximately 80 units of onabotulinum toxin A and follow-up evaluations were recorded at one, four, and five months after the injection. An average decrease of 2.03 in the DSFS sum score was recorded one month after injection, but the score returned to its baseline value four months after injection.

Two studies investigated the efficacy of BoNT-A as a treatment for sialorrhea in patients with amyotrophic lateral sclerosis (ALS). Paracka et al. observed a significant decrease in the Drooling Severity Scale at four, six, and twelve weeks after injection with incobotulinum toxin A, but the Drooling Frequency Scale only showed a statistically significant difference between the fourth and eighth week [22]. In a similar research work conducted by Rodriguez-Murphy et al., ten patients with ALS were injected with 40 units of onabotulinum toxin A, with 20 units at each side of the submandibular glands [21]. Posttreatment DSFS scores were calculated at four and twelve weeks after injection, and there was a noticeably significant difference between baseline and posttreatment values (*p* < 0.004).

Barbero et al. injected 250 units of abobotulinum toxin A into patients with sialorrhea secondary to neurological dysphagia [23]. Out of 38 patients participating in this study, 9 individuals were stroke patients, 8 patients were living with multiple sclerosis, 8 participants had Parkinson’s disease, 7 patients were living with amyotrophic lateral sclerosis, and 6 patients had Alzheimer’s disease. When the patients’ DSFS scores were assessed one and three months after injection, there was a statistically significant difference in the score from baseline values (*p* < 0.001).

Mazlan et al. performed a double-bind randomized trial studying the efficacy of different doses of abobotulinum toxin A in treating sialorrhea caused by neurological diseases [24]. Patients with different central nervous system diseases were assigned to injections with either 50 U, 100 U, or 200 U of abobotulinum toxin A, but there were no control groups implemented within the study. There was a decrease of 2 points in the DSFS score between the baseline and post-injection DSFS scores in all treatment groups. However, out of 29 patients who participated in the study (the “intent-to-treat” analysis), only 17 of them completed the entire trial (the “per-protocol” analysis). Although the researchers clearly explained that the results of both analyses are similar, they did not clarify whether the DSFS score they provided is from “intent-to-treat” or “per-protocol” analysis.

In brief, four of the seven studies included in the systematic review showed a statistically significant reduction in the DSFS score (*p* < 0.05) after injecting botulinum neurotoxin compared to the baseline [18,20,21,23]. Both trials by Nóbrega et al. and Santamato et al. had a similar study design as they included only patients with Parkinson’s disease and assessed the change in DSFS score one month after intervention. Nóbrega et al. and Santamato et al. reported a DSFS score reduction of 1.71 and 2.03, respectively. Rodriguez-Murphy et al.’s research showed a DSFS score reduction of 4.5 and 4.0 when assessing their patients four and twelve weeks after injection, respectively. Mazlan et al. separated their patients into three groups, each giving the patients different doses of abobotulinumtoxin A. In the group receiving 50 U, at six weeks post-injection, a clinically significant DSFS score reduction, defined by a decrease of 2 points in the DSFS score from the baseline, was observed. Patients that received 100 U displayed a 2-point DSFS score reduction six, twelve, and twenty-four weeks after injection. Only patients in the 200 U group displayed a 2-point DSFS score reduction two weeks post-injection. However, Mazlan et al. and Pal et al. did not mention whether this DSFS score reduction was statistically significant.

### 3.5. Safety and Adverse Events

A total of 298 patients were assessed for treatment-related adverse effects in the ten studies included in the meta-analysis and systematic review. Dry mouth was the most frequently reported adverse event, affecting 16 patients (5.37%). Fifteen patients reported mild and transient pain near the injection site, but the pain usually subsided without further treatment. The incidence of viscous saliva and difficulty in oral motor control was only observed in two patients (0.67%) and one patient (0.34%), respectively. Dysphagia, characterized by difficulty swallowing, was reported by two patients (0.67%). A speech disorder and eyelid ptosis were experienced by one patient (0.34%).

Some adverse events, although rare, necessitated careful monitoring and intervention. Bilateral local edema was found in one patient, and local bleeding was documented in one patient, both of which were promptly managed without further complications. One of the patients appeared to be depressed and forgetful and demonstrated signs of early dementia. Another patient who had a history of chronic bronchitis and bronchiectasis developed profuse mucopurulent coughing after the injection.

The risk of adverse events associated with the treatment was low, and the adverse effects usually persist under 24 h without intervention. However, the studies reported a higher chance of developing side effects from higher doses of botulinum toxins.

## 4. Discussion

This study aimed to assess the efficacy of BoNT-A in adults with drooling associated with central nervous system disorders. We concluded that it is safe and effective. Few meta-analyses have focused on the efficacy of BoNT-A in the treatment of various neurological disorders at the time of our study. One study determined the impact of botulinum toxin on sialorrhea in patients of any age diagnosed with any disease [25]. Other studies discovered the efficacy of botulinum toxin in the treatment of specific diseases, such as Parkinson’s disease [15,26]. Most meta-analyses primarily focused on BoNT-A injection for treating drooling in children with cerebral palsy [27]. This analysis found superiority of BoNT-A over placebo in the improvement of drooling severity and frequency in adult patients with central nervous system diseases.

The randomized controlled trial conducted by Narayanaswami et al. was the only study that did not find a significant change in DSFS score between the treatment and control groups. This could be due to researchers injecting a higher amount of incobotulinum toxin A into the submandibular gland (30 units) compared to the parotid gland (20 units). A meta-analysis on a similar topic suggested the potential benefit of higher doses of incobotulinum toxin A injected into the parotid glands [15]. However, this study had a relatively small sample size of only nine patients, limiting the demonstration of treatment efficacy.

The injection site of BoNT-A remains a subject of debate, with conflicting findings from various studies. In this meta-analysis, we found that the research of Narayanaswami et al. was the only study to inject a higher dose of BoNT-A in the submandibular gland than in the parotid gland. This might explain the lack of a significant difference in the post-injection DSFS score between the treatment and control groups. Meanwhile, another study on *Parkinsonism and related disorders* suggested that injection in the submandibular gland may be marginally more effective than injection in the parotid gland, but the difference in effectiveness was not significant [28]. Rodriguez-Murphy et al. injected 40 units of onabotulinum toxin A into the submandibular glands, but the submandibular glands were chosen as the site of injection because they were responsible for most of the salivary basal flow in tube-fed patients [21]. Given these points, current research has not reached a consensus on which salivary glands yield the best outcomes when injected with botulinum toxin.

There are three types of botulinum toxin type A products with different potencies in the studies included for our meta-analysis: onabotulinum toxin A, abobotulinum toxin A, and incobotulinum toxin A. Although the conclusions regrading efficacies from the various research works are similar, the dose equivalent unit ratio of onabotulinum toxin A/abobotulinum toxin A/incobotulinum toxin A was 1:2–3:1 [29]. This point should be taken into consideration for future studies of similar topics. Based on the available studies, higher doses of BoNT-A generally lead to a more significant decrease in DSFS score, particularly within a short period of time [15,26]. Mancini et al.’s study utilized 450 units of abobotulinum toxin A. Even with a conversion ratio of 3:1, 450 units of abobotulinum toxin A is equal to 150 units of incobotulinum toxin A. According to the forest plot, Mancini et al.’s study used the highest dose and had the greatest standardized difference in means. However, the assessment was conducted one week after injection, which is relatively early compared to other studies. The authors mentioned a potential one-month duration of abobotulinum toxin A’s effect, but did not provide the post-injection DSFS score [16]. Additionally, 100 units of incobotulinum toxin-A appeared to result in a slightly greater reduction in DSFS score compared to 75 units.

Although this meta-analysis successfully demonstrated the significant reduction in drooling severity and frequency with BoNT-A, several limitations were encountered—first, the small number of included studies in the meta-analysis. This was primarily due to the specific topic and inclusion criteria, resulting in limited recruitment. Only the Drooling Severity and Frequency Scale was used as the main outcome measure, as various studies employed different primary and secondary measures to assess drooling severity. Second, drooling can be commonly seen in patients with cerebral palsy, but most patients who are diagnosed with cerebral palsy were under 18 years old. Relevant studies with patients less than 18 years old were excluded in this research. Third, other types of botulinum toxins except BoNT-A were excluded. Combining different types of botulinum toxin may introduce heterogeneity due to variations in dosage and formulations. However, it is worth noting that Narayanaswami et al. conducted a meta-analysis that included both type A and type B botulinum toxins. A total of six studies were included in their meta-analysis, but a high level of heterogeneity (*I*^2^ = 49%) and a *p*-value of 0.083 were reported [15].

The optimal method and protocol for administering BoNT-A to adult patients who exhibit drooling associated with central nervous system diseases remains unknown and requires further investigation. Nonetheless, this study demonstrated more evidence supporting the use of BoNT-A in drooling. This review can provide considerable research directions for future studies.

## 5. Conclusions

In conclusion, injection of botulinum toxin type A into the salivary glands can be an effective and safe treatment for drooling in adult patients with central nervous system diseases. This meta-analysis demonstrates that there is a statistically significant difference in standardized difference in the means of DSFS scores between the treatment group and the control group. The qualitative data from within-subject studies (i.e., studies without a control group) show a significant difference between baseline and post-injection DSFS scores. Furthermore, only a few adverse events are reported. The amount of botulinum toxin and the injection site may affect the overall treatment outcome. Further research with the design of randomized control trials and large numbers of participants is necessary to reach a more accurate conclusion.

## Figures and Tables

**Table 1:** The Drooling Severity and Frequency Scale (DSFS) by Thomas-Stonell and Greenberg.

Severity Scale		Frequency Scale	
Definition	Score	Definition	Score
Dry: never drools	1	Never drools	1
Mild: only the lips are wet	2	Occasionally drools	2
Moderate: wet on the lips and chin	3	Frequency drools	3
Severe: drools to the extent that clothing becomes damp	4	Constantly drools	4
Profuse: clothing, hands, tray, and objects become wet	5		

**Table 2:** Basic characteristics of each study included in the meta-analysis.

Authors (Year)	Sample Size (Treatment; Control)	Mean Age (SD)	Number of Male and Female Participants (Male; Female)	Diagnosis	Botulinum Toxin Type a Used (Total Dose in Each Side of Parotid Gland [PR] and Submandibular Gland [SM])	Time of Assessment
Narayanaswami et al. (2016) [15]	9 (5; 4)	68.1 (9.7)	6; 3	Parkinson’s disease	IncobotulinumtoxinA, Xeomin (100 U total; 20 U in PR, 30 U in SM)	1 month
Mancini et al. (2003) [16]	20 (10; 10)	69.6 (6.1)	11; 9	Parkinson’s disease, multiple system atrophy	AbobotulinumtoxinA, Dysport (450 MU total; 141.65 MU in PR, 78.75 MU in SM)	1 week
Jost et al. (2019) [17]	184 (74 in each treatment group; 36 in the control group)	65.2 (11.4)	132; 54	Parkinson’s disease, stroke, traumatic brain injury	IncobotulinumtoxinA, Xeomin (75 U and 100 U in each of the two treatment groups. In the 75 U treatment group, 22.5 U is injected into the parotid gland, and 15 U is injected into the submandibular gland, per side. In the 100 U treatment group, 30 U is injected into the parotid gland, and 20 U is injected into the submandibular gland, per side.)	4 weeks, 8 weeks, 12 weeks, 16 weeks

**Table 3:** Basic characteristics of each study included in the systematic review.

Authors (Year)	Number of Male and Female Patients (Male; Female)	Mean Age (SD)	Diagnosis	Botulinum Toxin Type a Used (Total Dose in Each Side of Parotid Gland [PR] and Submandibular Gland [SM])	Time of Assessment	Mean Changes (Reduction in DSFS Score)	Number of Responding Patients
Nóbrega et al. (2007) [18]	18; 3	70 (ranging from 55–84 years)	Parkinson’s disease	AbobotulinumtoxinA, (250 U; 125 U at two points of PR)	1 month	1.71	19 (90.5%)
Pal et al. (2000) [19]	6; 3	75.2 (8.1)	Parkinson’s disease	OnabotulinumtoxinA, (First phase: 7.5 MU in PR, second phase: 15 U in PR)	8 weeks, 16 weeks	1.75 (8 weeks), 0.85 (16 weeks)	6 (66.7%)
Santamato et al. (2008) [20]	14; 4	71 (6.1)	Parkinson’s disease	OnabotulinumtoxinA, (82.0 ± 14.4 MU total, varies from 60~100 MU)	1, 4, and 5 months	2.03 (1 month)	18 (100%) (only in one month)
Rodriguez-Murphy et al. (2011) [21]	4; 6	68.5 (12.2)	Amyotrophic lateral sclerosis (ALS)	OnabotulinumtoxinA, (40 U; 20 U in SM)	4 weeks, 12 weeks	4.5 (4 weeks), 4.0 (12 weeks)	N/A
Paracka et al. (2019) [22]	8; 6	55.4 (16.3)	ALS	IncobotulinumtoxinA, Xeomin (150 MU; 50 MU in PR, 25 MU in SM)	4, 8, and 12 weeks	N/A	14 (100%)
Barbero et al. (2015) [23]	23; 15	68.9 (10.4)	Neurological dysphagia	AbobotulinumtoxinA, Dysport (250 IU; 75 IU in PR, 50 IU in SM)	1 and 3 months	3.96 (in 1 and 3 months)	N/A
Mazlan et al. (2015) [24]	20; 10	56 (16.1)	Stroke, traumatic brain injury	AbobotulinumtoxinA, (50 U, 100 U, 200 U in three treatment groups; each gland injected equal amount)	2, 6, 12, and 24 weeks	2.1 (50 U in 6 weeks), 2.1 (100 U in 6 weeks,), 2.8 (200 U in 6 weeks)	N/A

## Data Availability

Data are contained within this article.
